# Microbiome analysis of healthy and diseased sponges *Lubomirskia baicalensis* by using cell cultures of primmorphs

**DOI:** 10.7717/peerj.9080

**Published:** 2020-05-26

**Authors:** Lubov Chernogor, Elizaveta Klimenko, Igor Khanaev, Sergei Belikov

**Affiliations:** Limnological Institute of the SB RAS, Irkutsk, Russia

**Keywords:** *Lubomirskia baikcalensis*, Primmorphs, Symbionts, Opportunistic pathogen

## Abstract

Endemic sponges (Demosponges, Lubomirskiidae) dominate the fauna of the littoral zone of Lake Baikal. These freshwater sponges live in symbiosis with diverse eukaryotes and prokaryotes, including chlorophyll-containing microalgae. Within the last 5 years, the incidence of sponge disease and mortality events in Lake Baikal has increased. The etiology and ecology of these events remain unknown, in part because of the lack of models to study sponge-microbe interactions. In this work, we tested the use of primmorph cell cultures of *Lubomirskia baicalensis* as a tool for investigating the microbiomes of sponges. We infected primmorphs, cultured in vitro, with samples from diseased sponges and observed, by microscopy, disease symptoms, including loss of green symbionts, associated with mass die-off events. Subsequent sequencing of 16S rRNA gene fragments revealed that the microbiome community of healthy sponge and primmorphs formed a group separate from the community of diseased sponges and infected primmorphs. This confirms the suitability of the primmorph cell culture as a model sponge system. We also discovered mass mortality of green symbionts (Chlorophyta) was associated with a shift in the microbial communities of sponges/primmorphs. Microbes in diseased sponges, and infected primmorphs, belonged mainly to the phyla *Bacteroidetes* and *Proteobacteria* and these families *Flavobacteriaceae*,* Burkholderiaceae*, and *Moraxellaceae*. Primmorphs cell culture may provide a model to study interactions between these bacteria and their host and elucidate the cause of mass mortality events.

## Introduction

Sponges (phylum Porifera) are ancient multicellular animals that have existed for more than 635 million years and are unique in comparison to other Metazoa (Love et al., 2009). These sessile invertebrates filter vast volumes of water and convert dissolved organic matter into food for other animals. Metabolites from the sponge and their symbionts support both partners (Taylor et al., 2007; Fan et al., 2012; De Goeij et al., 2013). Sponges also indicate the state of the environment and play an important role in aquatic ecosystems (Wilkinson & Fay, 1979; Webster, 2007; Wulff, 2007; Bell, 2008) and are potential sources of biologically active molecules, which are extremely important for biomedicine and pharmacology because of their anticarcinogenic, antiviral, and antibacterial properties (Tsujii et al., 1988; Munro et al., 1999; Shakeri & Sahebkar, 2015). Most species of sponges are marine, while freshwater sponges are much less diverse.

Endosymbionts can account for more than half of the volume of sponges. These microbes influence their host metabolism (Bayer, Schmitt & Hentschel, 2007; Webster & Taylor, 2012). Sponges can maintain highly diverse, specific symbiont communities despite the constant influx of water microorganisms through the process of filter-feeding (Thomas et al., 2016; Moitinho-Silva et al., 2018). Sponge symbionts consist of a core microbiome, found in most sponge species, and a species-specific microbiome, consisting of narrow specialists, which differ in their relative numbers and rarely occur in other species (Thomas et al., 2016; Moitinho-Silva et al., 2018).

In recent years, disease and mass mortality of sponges and corals in the marine environment have been observed worldwide (Webster et al., 2004; Olson, Gochfeld & Slattery, 2006; Hoegh-Guldberg et al., 2007; Miller & Richardson, 2011; Pita et al., 2018). The die-off events threaten the entire sponge-associated biodiversity (Olson, Gochfeld & Slattery, 2006; Webster, 2007; Stabili et al., 2012). These changes in sponge-microbe interactions appear associated with climate change and the occurrence of opportunistic infections resulting from changes in water temperature caused by global warming, light intensity, and salinity (Webster et al., 2008; Luter, Whalan & Webster, 2010; Sagar, Kaur & Minneman, 2010; Erwin et al., 2012; Fan et al., 2013; Fujise et al., 2014).

Disease and mortality events threaten the freshwater sponges of Lake Baikal. Lake Baikal is located in southeastern Siberia (53°30′N 108°0′E) and is the world’s largest (23,000 km), deepest (1,643 m), and oldest (>24 million years) freshwater body (Kozhova & Izmest’eva, 1998). The lake has many features inherent to the ocean: abyssal depths, a huge mass of water, oxygen-rich water that goes to the very bottom, internal waves and seiches, strong storms and high waves, and upwelling (Kozhov, 2013; Smirnov et al., 2014; Troitskaya et al., 2015). Endemic freshwater sponges (Demosponges, Lubomirskiidae) dominate in Lake Baikal in the littoral zone at depths of 3 to 35 m. They cover close to 50% of the available surfaces (Pile et al., 1997) and represent a complex consortium of many species of eukaryotes and prokaryotes (Sand-Jensen & Pedersen, 1994; Bil et al., 1999). Sponges are chlorophyll-containing freshwater organisms due to their association with various chlorophyll-producing algae (Latyshev et al., 1992; Bil et al., 1999). These photosynthetic parthers give healthy sponges in the photic zone of Lake Baikal a green color (Wilkinson & Garrone, 1980; Alex et al., 2012; Chernogor et al., 2013; Raharinirina, Brandt & Agostino Merico, 2017).

Anomalously pink-colored *L. baicalensis* (Pallas, 1776) sponges first appeared in Lake Baikal in 2011. Now diseased sponges occur throughout the littoral zone of the lake with various symptoms of damage to the body, such as discoloration, tissue necrosis, the formation of brown plaques and dirty-violet bacterial covers on separate branches. Annually, up to 10–20% of diseased sponges die each winter (Timoshkin et al., 2016; Khanaev et al., 2018). Diseased and dead sponges have been observed in many areas of the lake (Kravtsova et al., 2014; Khanaev et al., 2018; Belikov et al., 2019). Researchers noticed a large-scale disturbance in the spatial distribution and structure of phytocoenoses in the coastal zone of Lake Baikal (Timoshkin et al., 2016). Some authors have described the bacterial communities in diseased sponges, but no pathogenic agents have been identified (Kaluzhnaya & Itskovich, 2015; Kulakova et al., 2018). The etiology and ecology of the disease and mass death of sponges remain unknown.

The development of a model to investigate the transmission of pathogenic agents from diseased sponges requires a detailed study of pathogen-host interactions in the environment. However, these experiments with sponges are difficult to perform under the natural conditions of Lake Baikal. In our study, we experimentally infected cell cultures of primmorphs in vitro and used targeted metagenomic analysis of PCR-amplified 16S rRNA genes to establish primmorphs as model for sponge-microbiome-disease interactions.

## Material and Methods

### Sponge collection and cell culture of primmorphs

**Figure 1:**
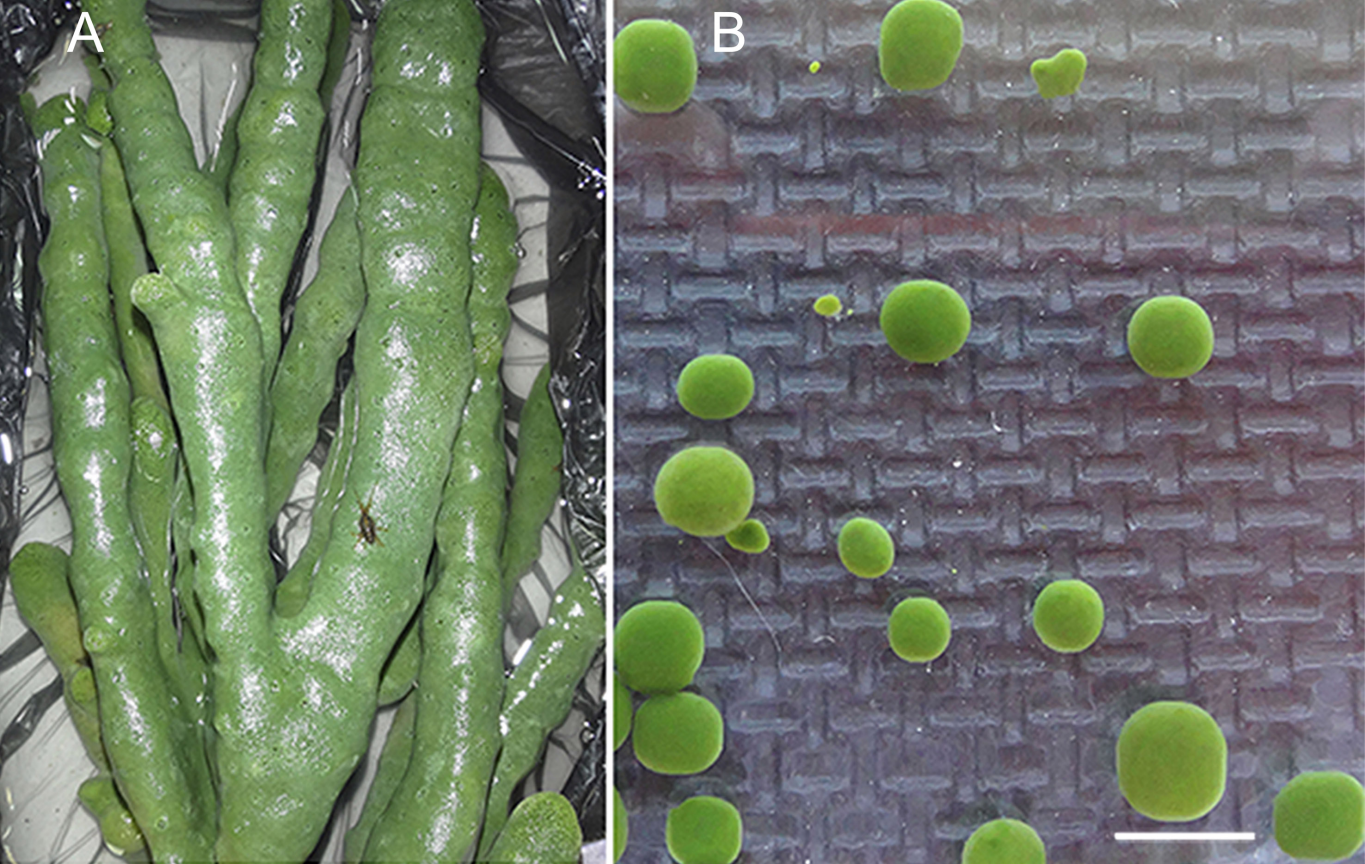
Samples of the healthy sponge and primmorphs. (A) The healthy freshwater Baikal sponge *L. baicalensis*, (B) cell culture of primmorphs of *L. baicalensis* obtained from the sponge. Scale bars are 5 mm. Photographs taken with Canon EOS 200D digital camera.

The freshwater Baikal sponge *L. baicalensis* Pallas, 1776 (Demospongiae, Haplosclerida, Lubomirskiidae) was the object of this study (Figs. 1A and 1B). Specimens were collected in individual containers from Lake Baikal in the Olkhon Gate Strait (53°02′21N″; 106°57′37E″) at a depth of 10 m (water temperature 3–4°) by scuba divers in February 2015. The collected samples of sponges were immediately placed in containers with Baikal water and transported to the laboratory. We selected sponges without visible symptoms of the disease and those colored green (Figs. 1A and 1B). The group of healthy samples included healthy sponge and primmorphs cultivated for 1 and 14 days (Table 1).

**Figure 2:**
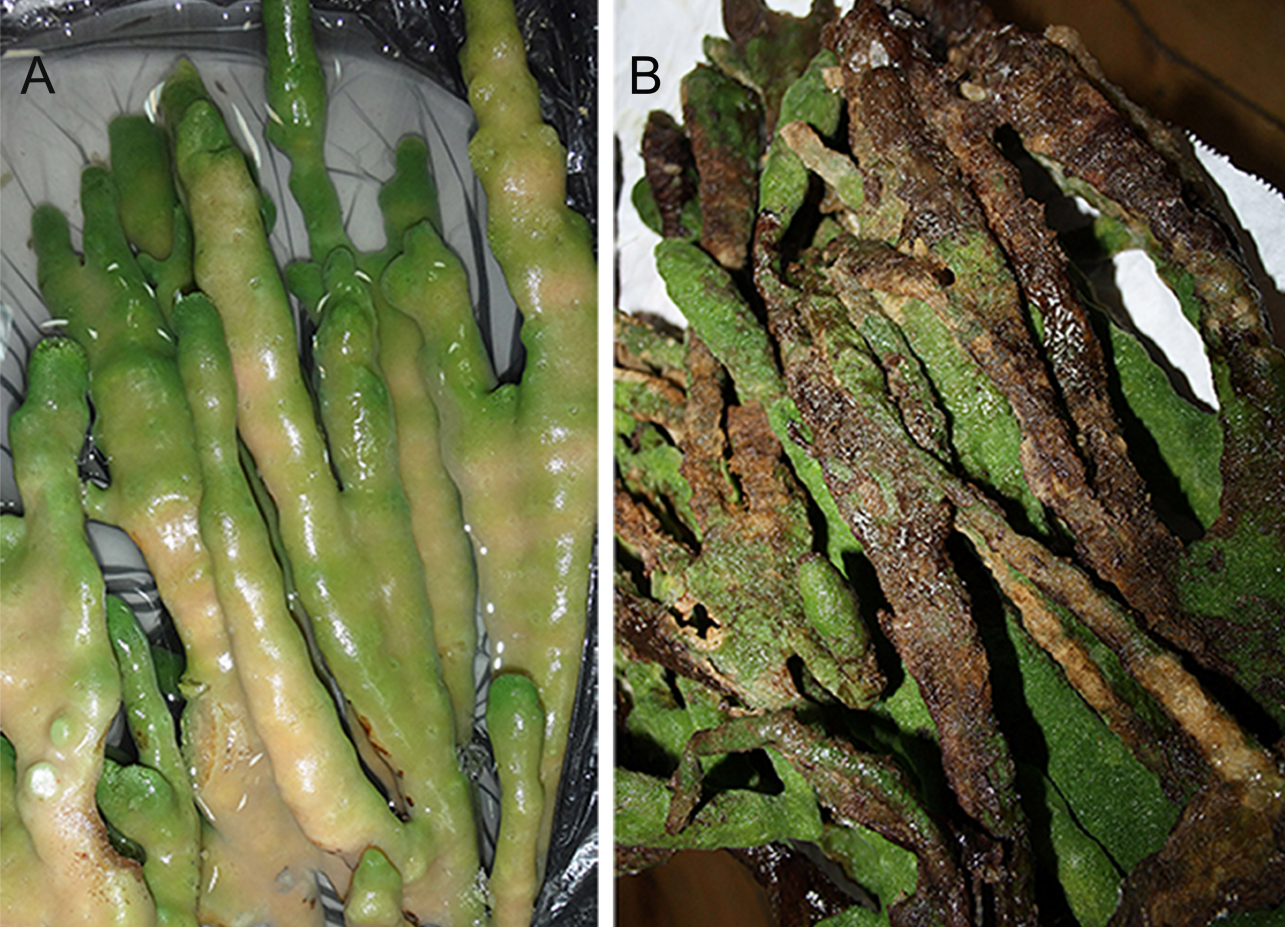
Samples of diseased sponges. (A) The diseased freshwater Baikal sponge *L. baicalensis*. (B) The sick freshwater Baikal sponge *L. baicalensis.* Photographs taken with Canon EOS 200D digital camera.

Samples of diseased and sick sponges were collected to obtain a bacterial suspension to infect healthy cell cultures of primmorphs. The group of diseased samples included diseased and sick sponges and experimentally infected primmorphs, as well as primmorphs obtained from these diseased sponges (Table 1 and Figs. 2A and 2B). Primmorphs were obtained by mechanical dissociation of cells according to the previously described technique (Chernogor et al., 2011). A clean sponge was squashed, and the obtained cell suspension was subsequently filtered through a sterile 200-, 100-, and 29- μm nylon mesh to eliminate pieces of skeleton and spicules of the maternal sponge. The gel-like suspension was diluted 10-fold with Baikal water, placed in a refrigerator, and stored for 5 min at 3–6 °C until a dense precipitate formed. The precipitate was then washed several times with sterile Baikal water until the complete elimination of the turbid uppermost layer. Natural Baikal drinking water (NBW) that was obtained at a depth of 500 m, passed through sterilizing filters and processed with ultraviolet light and ozone (Patent of the Russian Federation No. 2045478) was used for cultivation. The suspension was placed into 200–500-ml cultural bottles (Nalge Nunc International, Rochester, NY, USA) and washed with NBW twice every 30 min for the first 2 h. Primmorphs were cultivated in NBW at 3–4  °C and at a light intensity of 47 lux or 0.069 watts with a 12-hour day-night cycle.

**Table 1:** Samples of the sponge *L. baicalensis*. Samples were collected from the Olkhon Gate Strait, Lake Baikal.

**Coordinate**	**Sample ID**	**Description**
53°02′21″N	SH	Healthy sponge with bright green color
106°57′37″E	PH1	Primmorphs cultivation for 1 day
	PH14	Primmorphs cultivation for 14 days
	SD	Bleached sponge
	PD	Primmorphs diseased
	PID	Primmorphs infected by diseased sponge
	SS	Sick sponge with tissue necrosis
	PIS	Primmorphs infected by sick sponge

### Experimental infection of primmorphs

**Figure 3:**
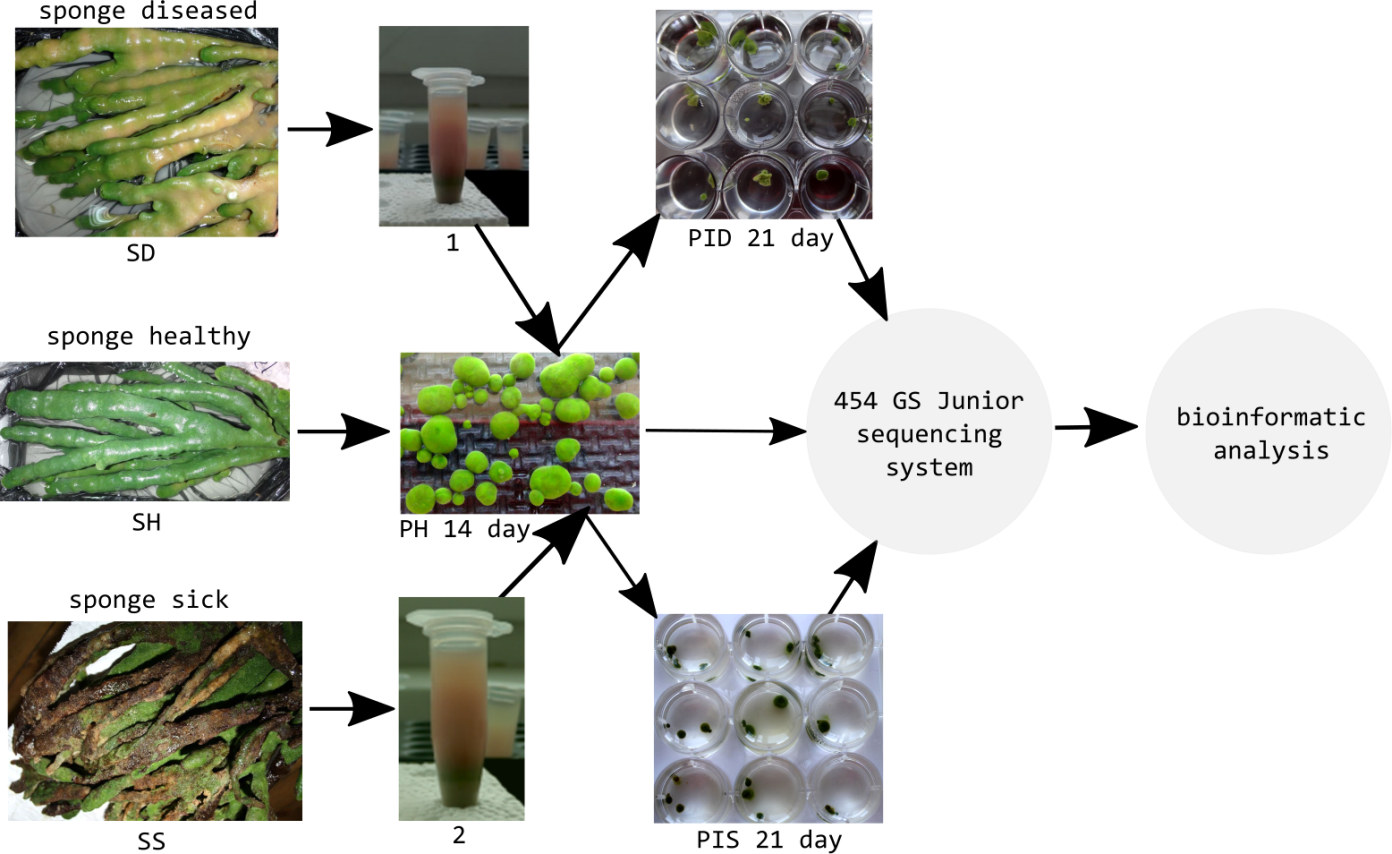
Experimental design study of the microbiomes of healthy, diseased sponges and primmorphs. 1, the suspensions of microorganisms from diseased sponge; 2, the suspensions of microorganisms from sick sponge.

Healthy cell cultures of primmorphs (2–4 mm in diameter) of green color were transferred to six 24-well plates (Nalge Nunc International, Rochester, NY, USA), 1–2 pieces per well. We used three 24-well plates for each experiment for infection of primmorphs with suspensions of microorganisms from diseased and sick sponges (Fig. 3). The suspensions of microorganisms were obtained by squeezing 10 g samples of diseased and sick sponges. The cell suspensions were purified by filtering through sterile 100- and 29- μm nylon meshes to eliminate pieces of skeleton and spicules from the maternal sponge and were subsequently filtered through 10- μm filters (Millipore, Germany). Then, the cell suspensions were diluted 10-fold with cold NBW, and cellular debris was removed by centrifugation at 1500 rpm for 3 min. Healthy primmorphs were infected with 25 μl of suspensions from diseased and sick sponges. Primmorphs were cultivated in 2 ml of NBW at 3–6 °C with a 12-hour day and night cycle for 30 days. During the infection, the observations were carried out with daily records, as well as for sampling, DNA isolation, and sequencing of microbiomes.

### Microscopy studies

We observed changes in infected and diseased cell cultures of primmorphs every day for 1 month with an Axio Imager Z 2 microscope (Zeiss, Germany) equipped with fluorescence optics (self-regulating, blue HBO 100 filter, 358/493 nm excitation, 463/520 nm emission). The samples of cell cultures were stained with a NucBlue Live ReadyProbes reagent (Invitrogen, Ambion, USA). All the images were taken with a Canon EOS 200D digital camera. In addition, the samples were prepared for scanning electron microscopy (SEM) analyses. Fixation was performed according to the following procedure: pre-fixation in 1% OsO_4_ (10 min), washing in cacodylate buffer (30 mM, pH 7.9) (10 min), fixation in 1.5% glutaraldehyde solution on cacodylate buffer (30 mM, pH 7.9) (1 h), washing in cacodylate buffer (30 mM, pH 7.9) (30 min), postfixation in 1% OsO_4_ solution on cacodylate buffer (30 mM, pH 7.9) (2 h), washing in filtered Baikal water for 15 min at room temperature, and dehydration in a graded ethanol series. The specimens were placed into SEM stubs, dried to a critical point, and coated with liquid carbon dioxide (BalTec CPD 030) using a Cressington 308 UHR sputter coater before examination under Sigma series scanning electron microscope (Zeiss, Germany) operated at 5.0 190 kV.

### DNA extraction, PCR amplification, and sequencing

DNA was extracted from the samples of sponge tissue (0.1–0.2 g) and primmorphs after bead beating using the TRIzol LS reagent (Invitrogen, USA) according to the manufacturer’s protocols. Total DNA from three technical replicates for each sample was suspended in 18 μl of RNase-free water and stored at −70 °C pending further analysis. The universal bacterial primers 518F and 1064R (Ghyselinck et al., 2013) were used to amplify the V4–V6 hypervariable region of the bacterial 16S rRNA gene. The following program was used to amplify 16S rRNA genes using PCR: 3 min at 96 ^∘^C; 30 cycles at 94 °C for 20 s, 55 °C for 20 s and 72 °C for 1 min with a final 10-min incubation at 55 °C. PCR products were quantified using the NanoDrop device, mixed equally and sequencing using the 454 GS Junior Sequencing System (Roche, Basel, Switzerland) with GS FLX Titanium series reagents. Raw sequencing data are available in the NCBI Sequence Read Archive under accession number PRJNA480187.

### Processing of sequencing data

Bioinformatics and statistical analyses were performed using the QIIME2 2019.1 pipelines (Bolyen et al., 2018). First, the reads were demultiplexed; then, they were subject to quality analysis to determine trimming parameters. The first 17 nucleotides for each read were trimmed; the total length of reads was truncated to 360 nucleotides due to the decrease in quality score observed after 360 nucleotides. For more consistent results, the reads containing any ambiguities were removed. After quality screening and trimming, the DADA2 pipeline was used to remove chimeric variants and to identify sub-OTUs (Callahan et al., 2016). Sub-OTUs are determined by the analysis of polymorphic sites within amplicons and show a greater taxonomic sensitivity than OTUs clustered by a 3% dissimilarity threshold (Callahan et al., 2016; Thompson et al., 2017). Compared to OTUs, the analysis of sub-OTUs has proven to be effective in resolving fine-scale ecological temporal dynamics and community changes in the human microbiome. This is precisely why we used sub-OTUs rather than OTUs in analyzing sponge and primmorph microbiomes (Eren et al., 2014; Tikhonov & Wingreen, 2015). The SILVA 132 database was used for taxonomic assignment. Reference sequences in SILVA 132 database were first trimmed to the V4–V6 region with the 518F/1064R primers used in the PCR. Taxonomy assignments were performed using q2-feature-classifier (Bokulich et al., 2018). Sequences identified as mitochondria were removed from libraries prior to the analysis. The relative abundance of mitochondrial reads across all libraries was <0.000001%, which had a minimal effect on the library size upon the removal. Sub-OTU relative abundance values were calculated by transformation to the library read depth. In total, eight libraries were analyzed. The alpha-diversity indices (Chao1, Shannon diversity index) were calculated using the QIIME software to establish the abundance and the diversity of the sequences. Weighted Unifrac dissimilarity values were used for β-diversity measurements (Lozupone & Knight, 2005). The Principal Coordinates Analysis (PCoA) (Halko et al., 2010) was used to visualize β-diversity, and the grouping variable significance (stage of life and health) was assessed using PERMANOVA pairwise test (Anderson, 2001). For interpretation of the microbial community, we used the tidyr R package and ggplot2 R package to build a heat map, from the 20 most abundant sub-OTUs.

## Results

### Microscopy studies of cell cultures of primmorphs

**Figure 4:**
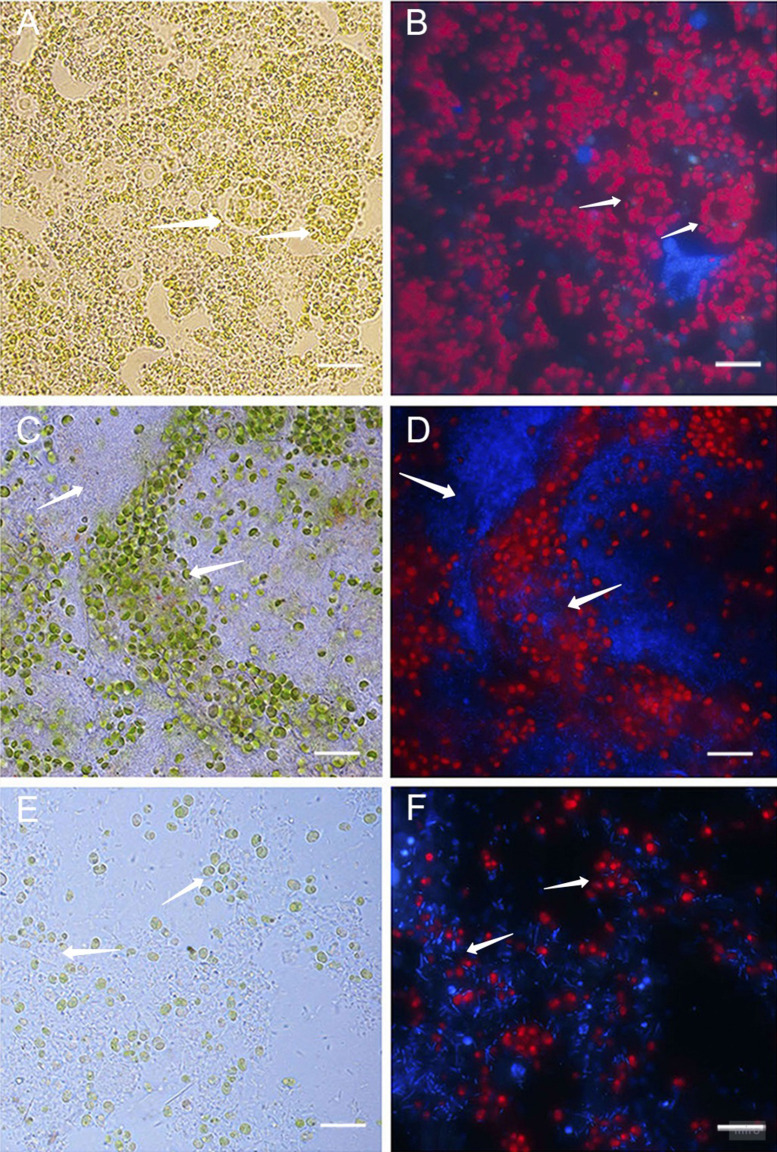
Light and fluorescence images of cell cultures of primmorphs of sponge *L. baicalensis.* (A) Light microscopy, shows green microalgae located within amoebocytes in the healthy cell culture of primmorphs. Arrows show sponge amoebocytes within microalgae (B) Fluorescence microscopy, shows red autofluorescence of chlorophyll-containing intracellular of green algae in the healthy primmorphs. (C) The primmorphs infected with cellular suspension from the diseased sponge observed the death of green algae symbionts, sponge cell (indicated by arrow). Huge numbers of different bacteria on day 7 are shown (indicated by arrow). (D) Fluorescence microscopy shows the death of microalgae (red color) in infected primmorphs from diseased sponge on day 7. Bacteria are shown with blue color. (E) The primmorphs infected with cellular suspension from the diseased sponge during 21 days, shown residues of green algae in cell culture of primmorphs and huge biomass of bacteria (indicated by arrow). (F) Fluorescence microscopy shows death of green algae primmorphs infected with the suspension from the diseased sponge and massive of different bacteria during 21 days. Bacteria in infected primmorphs are shown with blue color. The samples of primmorphs stained with the NucBlue Live ReadyProbes reagent for fluorescence microscopy. Scale bars: 10 µm.

Healthy cultures of primmorphs had a bright green color and bright red autofluorescence of chlorophyll in cells on the second and the following days of cultivation (Figs. 4A–4B). We observed different cells of the sponges, amoebocytes with nuclei and inclusion of green symbiotic algae were clearly visible. A completely different picture was observed in infected primmorphs. All samples of primmorphs lost green color after being infected with a suspension of microbial cells from diseased and sick sponges after 3–4 days. We observed an imbalance in cells of sponges, a chaotic arrangement of algae, destruction of their cell walls, and the increase in the number of bacteria of different types after 7 days (Figs. 4C and 4D).

After 21 days, there was a full loss of green algae and sponge tissues with the growth of different bacteria in primmorphs infected with a diseased sponge (Figs. 4E and 4F). A loss of chlorophyll autofluorescence was observed in all the experimental samples. Bleaching and death of the cell culture of primmorphs were observed after 21 days. A similar developmental of the infection dynamics was observed in the primmorphs infected with the suspension from sick sponge cells.

**Figure 5:**
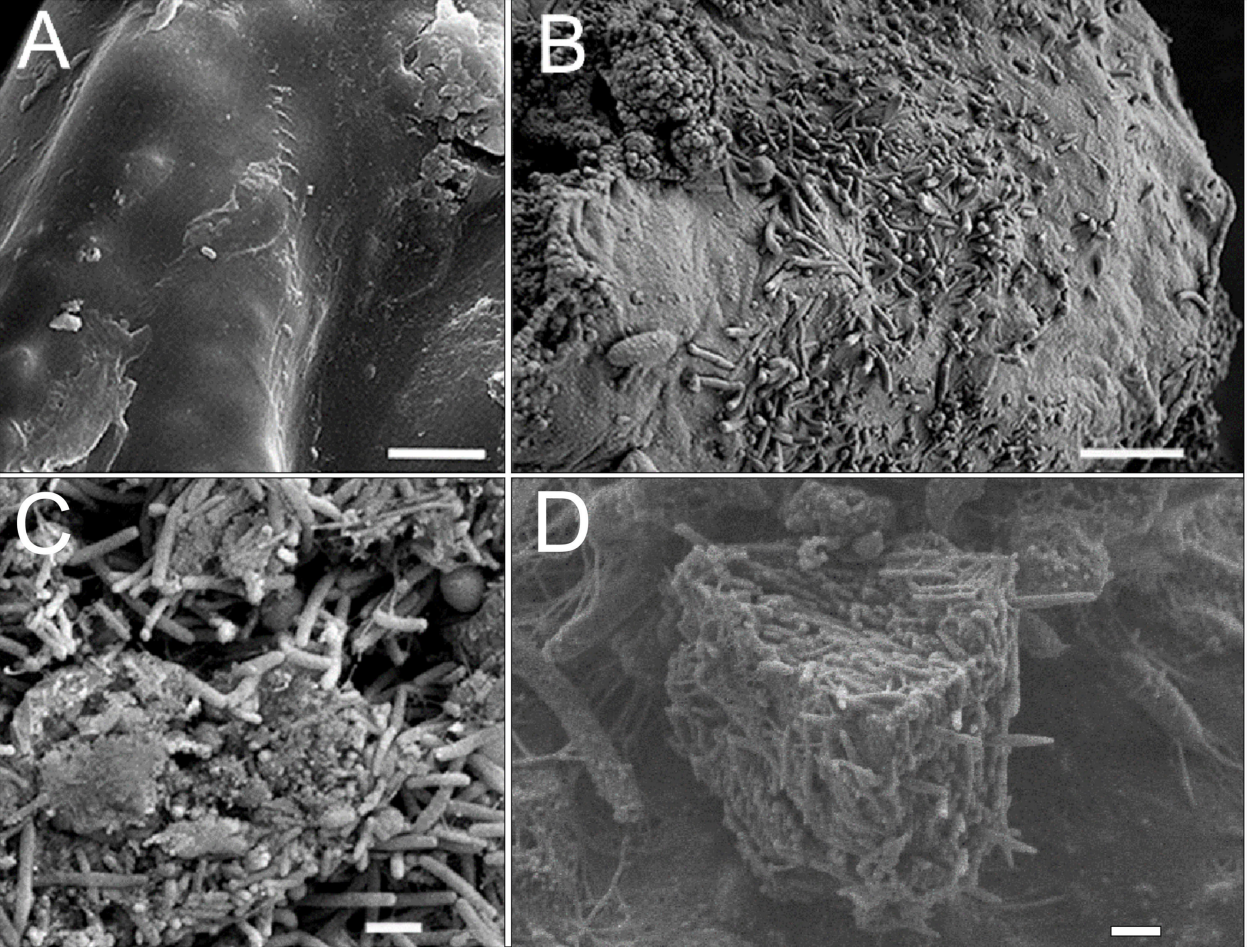
SEM images of cell cultures of primmorphs. (A) The epithelial surface of healthy cultures was clean, flat and smooth. (B) The surface of the primmorphs infected with the cellular suspension from the diseased sponge. The melting of the sponge epithelial cells and increase in different bacteria on day 7 were observed. (C) The primmorphs infected with cellular suspension from the diseased sponge, the death of green algae symbionts, sponge cells and massive growth of different bacteria for 21 day. (D) Bio-cake formed in infected cultures of primmorphs from diseased sponge on day 30. Scale bars are 1 µm.

Similar results were found in diseased and sick sponges and in the infected primmorphs. Dirty scurf, fetid odor, and formation of biofilms were observed in all the infected cultures, this was probably associated with the growth of different bacteria. The same pattern of disease development was observed when using SEM methods. In healthy primmorphs, the epithelium surface was clean, even, and smooth (Fig. 5A), whereas infected primmorphs had desquamated epithelium destroyed by different bacteria (Fig. 5B). As a result, the surfaces of infected primmorphs became uneven and eroded by numerous microorganisms penetrating the spongin, that lead to degradation, necrosis, and death of cells and tissues (Fig. 5C). We observed a huge amount of bacteria in infected cultures of primmorphs that formed a bio-cake after 30 days of cultivation (Fig. 5D). Moreover, there was a high increase in the biomass of different various bacteria accompanied by the death of cells and tissues of primmorphs.

### Samples description

Metagenomic analysis showed disease altered the diversity of the microbiome of primmorphs. The sum of reads for all the libraries passing the quality control parameters totaled 30,488 reads with a mean library depth of 3,811 reads/library. The number of reads ranged from 2155 in healthy primmorphs to 7564 in infected primmorphs. The estimates of sampling depth using Michaelis-Menten fit to rarefaction curves showed that the composition of microbiomes at the sub-OTU level was underestimated by 8.2% (Table 2).

**Figure 6:**
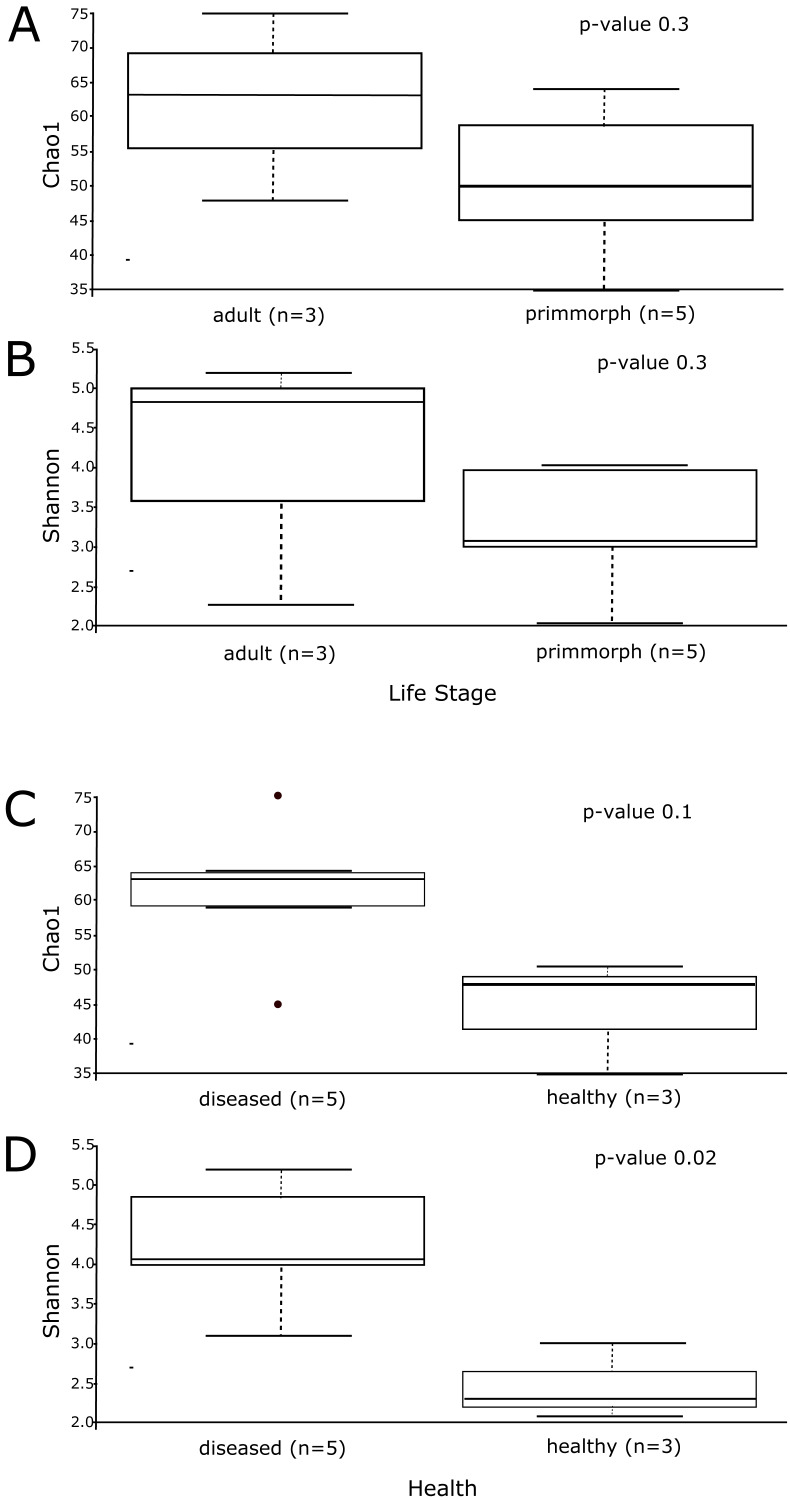
The alpha-diversity results of Chao1 and Shannon index shows the data distribution between the groups. (A) The distribution between the group’s adult sponge and primmorphs by Chao1 estimation. (B) The distribution between the group’s adult sponge and primmorphs by Shannon index. (C) The distribution between the groups of healthy and diseased sponges and primmorphs by Chao1. (D) The distribution between the groups of healthy and diseased sponges and primmorphs by Shannon index. Samples were referred to Table 1.

Alpha diversity was similar for healthy primmorphs, cultured in vitro, and healthy sponges, collected in the lake. The total number of sub-OTUs ranged from 35 to 61 in healthy sponges and primmorphs and from 43 to 76 in the diseased samples. Microbial diversity within each group was calculated and compared between the two groups. The microbial richness estimator (Chao 1 index values) showed no significant difference (Fig. 6A). Using the Shannon index, we did not find significant differences in the microbial diversity between the adult sponge and primmorphs (Fig. 6B). The alpha-diversity indices (Chao1 and Shannon index) had a significant difference between healthy and diseased groups (Table 3, Figs. 6C–6D).

**Table 2:** Pyrosequencing summary of microbial communities in sponges and primmorphs.

**Samples analysis data**	**Healthy group**			**Diseased group**				
****	**SH**	**PH1**	**PH14**	**SD**	**PD**	**PID**	**SS**	**PIS**
**Input**	2511	2449	2879	3340	7296	8064	3314	4969
**After clean**	2284	2155	2648	2379	6273	7564	2940	4331
**Sub-OTUs**	48	50	35	75	62	41	65	57
**Michaelis-Menten fit**	53.81	53.81	38.37	80.13	66.18	47.4	68.06	62.05

**Notes.**

The name of samples: SH (Healthy sponge); PH1 (Primmorphs for 1 day); PH14 (Primmorphs for 14 days); SD (Bleached sponge); PD (Primmorphs diseased); PID (Primmorphs infected by diseased sponge ); SS (Sick sponge); PIS (Primmorphs infected by sick sponge). Samples IDs are referred to Table 1.

**Figure 7:**
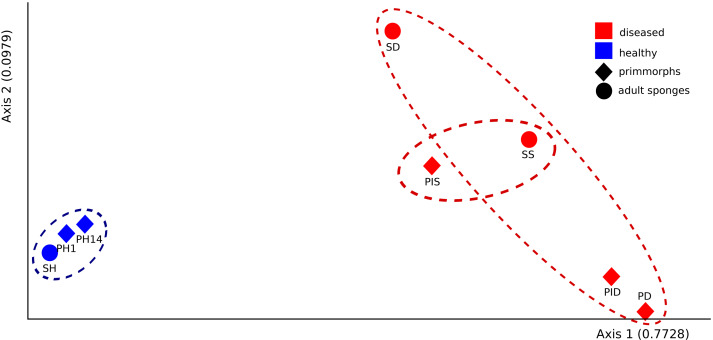
The beta-diversity results of PCoA indicating the data distribution between groups. Samples of the healthy sponge and primmorphs are grouped into one cluster and differ significantly from the group of the diseased ones. Samples were referred to Table 1.

The microbial communities of samples of diseased sponges and infected primmorphs were more diverse than healthy sponges and uninfected primmorphs. The Shannon index for healthy samples varied from 1.98 to 2.86, while this for the diseased samples was between 3.34 and 6.22 (*p* < 0.05) because of the high abundance of chloroplasts in healthy samples. The variation in data distribution between the groups was analyzed using the PERMANOVA test, which indicated a significant difference (*p* < 0.05) between healthy and diseased groups. PERMANOVA pairwise testing did not show any difference between the healthy sponge and the primmorphs cell culture (pseudo-F 0.4), while there was a significant difference between the healthy and the diseased group of samples (pseudo-F 13.8). Analysis of beta-diversity showed that microbiomes associated with healthy primmorphs were significantly (*p* < 0.05) more similar to microbiomes associated with healthy sponges, than with microbiomes associated with diseased organisms, and PCoA clearly distinguished healthy and diseased clusters (Fig. 7).

### Composition of the microbial community

#### Abundance and significant difference between the two groups at the phylum level

**Figure 8:**
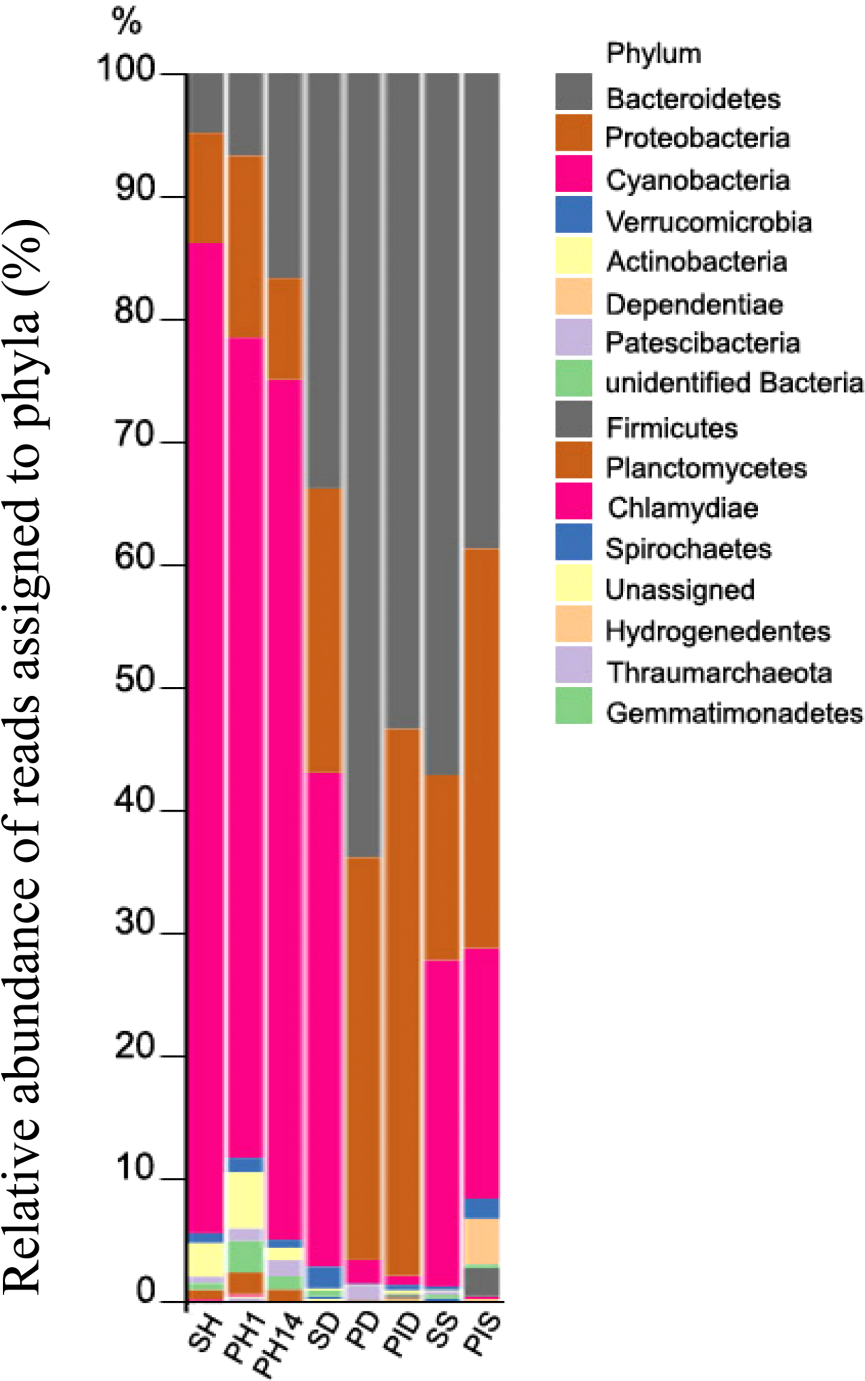
Taxonomic profiles of the microbial communities at the phylum level. Relative abundance of reads assigned to phyla (in %). Samples were referred to Table 1.

Five bacterial phyla comprised more than 96% of the community composition: *Bacteroidetes*, *Proteobacteria*, *Cyanobacteria*, *Actinobacteria*, *Verrucomicrobia*, and *Dependentiae* (Fig. 8). The healthy group of sponge/primmorphs was mainly composed of *Cyanobacteria*/chloroplast, while the diseased group contained *Bacteroidetes* and *Proteobacteria* (Fig. 8). The most significant differences were observed in the composition and structure of representatives of the taxonomic group *Cyanobacteria*/chloroplasts in microbiomes of diseased and healthy *L. baicalensis* sponges and cell cultures of primmorphs. The phylum *Cyanobacteria* dominated with abundances of 75–89% in healthy sponges and in cell cultures of primmorphs, while chloroplast abundance decreased to 10–20% in diseased sponges and to 0.7–22% in diseased and infected primmorphs. The most abundant bacteria at the phylum level were *Bacteroidetes*, with abundances of 48%, 72%, 65%, 55%, and 41% in the diseased group of sponges and primmorphs (Fig. 8). The other bacterial phyla were *Proteobacteria* with abundances 32%, 18%, 33%, 45%, and 33%, respectively. We found that *Alphaproteobacteria* dominated in healthy sponges and primmorphs, but they were replaced by *Betaproteobacteria* and *Gammaproteobacteria* in the diseased group. Also, an increase to 4% was found for *Dependentiae* (TM6) in primmorphs infected with a sick sponge.

**Table 3:** The alpha-diversity indices (Chao1, Shannon index). The alpha-diversity was calculated using the QIIME2 software to establish the abundance and diversity of the sequences.

**Samples ID**	**Sub-OTUs**	**Chao1**	**Shannon index**
**PH1**	50	51.00	3.01
**PH14**	35	35.00	2.04
**PD**	62	64.00	4.16
**PID**	41	45.00	3.09
**PIS**	57	59.00	3.90
**SH**	48	48.00	2.30
**SD**	75	76.00	5.21
**SS**	65	65.00	4.88

**Notes.**

The samples IDs were referred to Table 1.

#### Abundance and significant difference between the two groups at the family level

**Figure 9:**
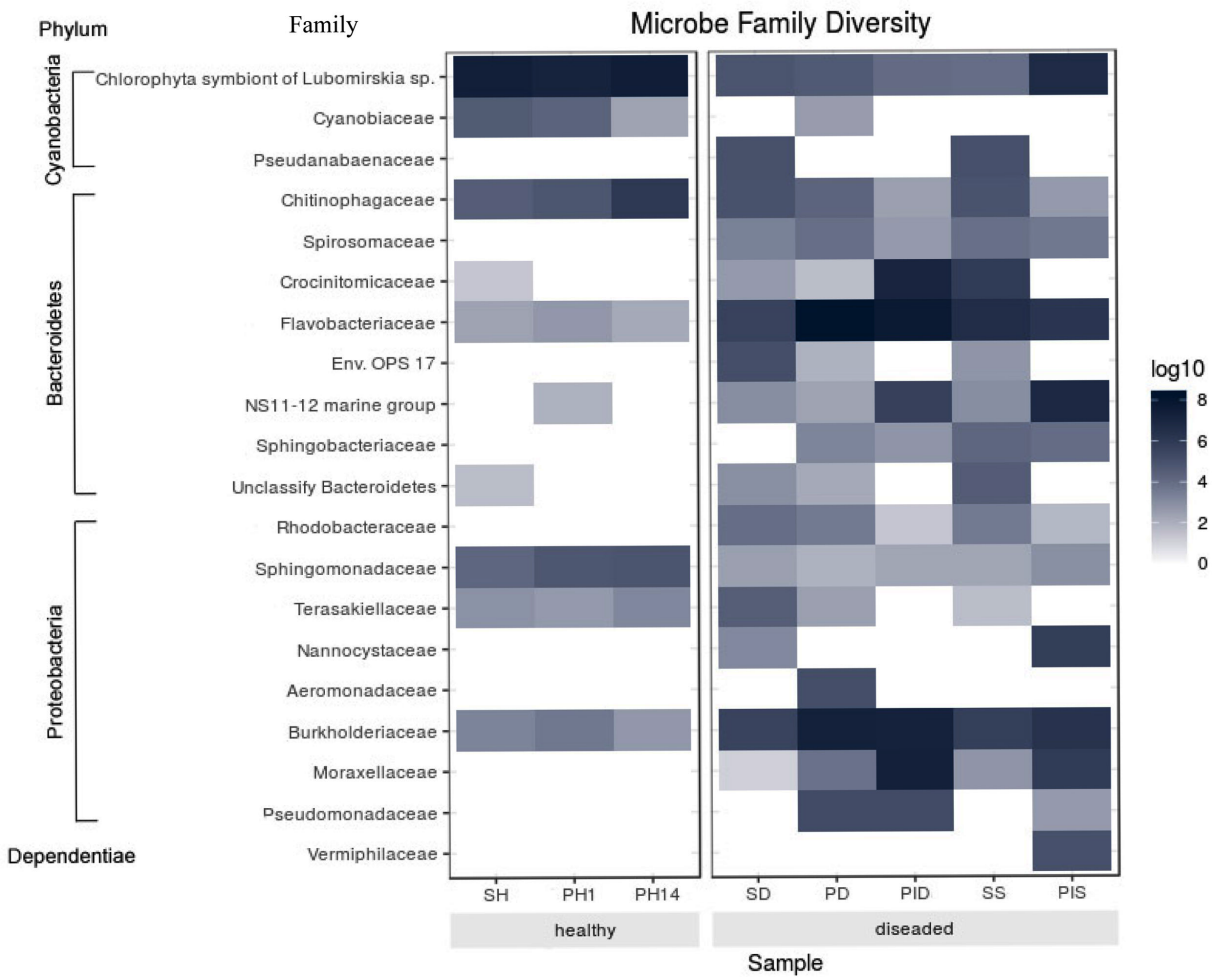
Heatmap shows the family with significant differences of relative abundances amongst the two groups. Sample IDs are referred to Table 1. The heatmap is based on the scale of 0–8 log.

The most abundant microorganism at the family level was chloroplast, homologous to the Chlorophyta symbiont of *Lubomirskia* sp. (Fig. 9). This symbiont dominates in healthy sponges and primmorphs with abundances of 84%, 77%, and 75% (Table 4). The family *Cyanobiaceae* was present in the healthy sponge (5%) and primmorphs (4–0.4%). They decreased with the cultivation time, *Pseudabaenaceae* were present in diseased sponges (11%). Moreover, the family *Flavobacteriaceae* should be noted to be significantly more abundant (13% to 62%) in the diseased sponges and primmorphs (Table 4). The other abundant family of bacteria was *Crocinitomicaceae* (17% in PID and 18% in SS). We detected an increased relative abundance in the *Sphingobacteriales* NS11-12 marine group in infected primmorphs (4% in PID, 26% in PIS) and an uncultured eubacterium env. OPS 17 was found in diseased sponges only (12%). Representatives of the *Chitinophagaceae* family were mainly found in the healthy group of sponges and primmorphs (5–18%) and in diseased sponges (6–10%), but they were not found in infected primmorphs. Our experimental results showed that the family * Alphaproteobacteria* had a high abundance in the healthy group of sponge and primmorphs. The family *Sphingomonadaceae* was abundant (3–7%) in the healthy group, but not in the diseased group. *Terasakiellaceae* and *Burkholderiaceae* were not well represented (0.5–2%) in the healthy group but were highly abundant in the diseased groups (14%, 15%, 19%, 20%, and 26%, respectively). Representatives of the families *Moraxellaceae* (10–22%) and the *Pseudomonadaceae* (0.3–3%) were found in diseased and infected primmorphs. Moreover, *Nannocystaceae* were found in the diseased sponge and primmorphs (up to 9%). *Vermiphilaceae* were found (4%) only in primmorphs infected with the diseased sponge.

## Discussion

Here, we investigated the possibility of using the cell culture of primmorphs *L. baicalensis* for the examination of microbiomes of diseased sponges and show the transmission of microorganisms from diseased sponges to healthy primmorphs. We found that the use of primmorph cultures model healthy sponges well. Both the alpha and beta diversity indices had no differences between the groups of adult sponge and primmorphs. The microbial communities of healthy sponges and primmorphs grouped separately from the communities of diseased sponges and infected primmorphs, which confirms the suitability of the cell culture of primmorphs as a model sponge system.

**Table 4:** Abundance and significant difference between the two groups at the phylum/family level.

**Phylum**		**Family**	Healthy group			**Diseased group**				
	****	****	**SH**	**PH1**	**PH14**	**SD**	**PD**	**PID**	**SS**	**PIS**
**Cyanobacteria**		Chlorophyta symbiont of *Lubomirskia* sp.	84.15	77.3	74.85	9.34	1.76	0.76	2.41	21.9
		Cyanobiaceae	5.04	4.43	0.41	0	0.2	0	0	0
		Pseudanabaenaceae	0	0	0	10.63	0	0	7.32	0
**Bacteroidetes**		Flavobacteriaceae	0.48	0.8	0.32	20.04	62.04	32.56	36.47	12.67
		Crocinitomicaceae	0.15	0	0	0.93	0.07	17.32	18.03	0
		NS11-12 marine group	0	0.34	0	1.36	0.16	4.13	0.88	25.55
		Chitinophagaceae	4.56	7.18	17.61	10.34	1.19	0.15	6.44	0.32
		Spirosomaceae	0	0	0	2	0.83	0.18	2.27	0.92
		Sphingobacteriaceae	0	0	0	0	0.41	0.2	3.1	1.36
		Env. OPS 17	0	0	0	11.91	0.1	0	0.7	0
		Unclassify Bacteroidetes	0.19	0	0	1.28	0.13	0	4.54	0
**Proteobacteria**	**α**	Rhodobacteraceae	0	0	0	3.71	0.57	0.04	1.62	0.12
****	****	Sphingomonadaceae	3.34	7.01	5.31	0.78	0.1	0.12	0.42	0.45
****	****	Terasakiellaceae	0.82	0.75	0.93	6.63	0.18	0	0.19	0
****	**β**	Burkholderiaceae	1.26	2.18	0.57	19.33	25.65	19.79	14.92	14.26
****	**γ**	Moraxellaceae	0	0	0	0.14	0.75	22.19	0.7	9.57
****	****	Pseudomonadaceae	0	0	0	0	3.07	2.56	0	0.32
****	****	Aeromonadaceae	0	0	0	0	2.8	0	0	0
****	**δ**	Nannocystaceae	0	0	0	1.57	0	0	0	8.63
**Dependentiae**		Vermiphilaceae	0	0	0	0	0	0	0	3.92

**Notes.**

(in %). Lines in bold type indicate a shift in microbial communities in the healthy and diseased groups.

The microbial community of a healthy group of sponge/primmorphs was mainly composed of the phylum *Cyanobacteria* (*Chlorophyt* a symbiont of *Lubomirskia* sp.) Baikal sponges, and other freshwater sponges, is their ability to live in symbiosis with various zoochlorellae (Bil et al., 1999). We previously showed that the primary photosynthetic algae belonging to green algae of the order Chlorophyta dominate in healthy *L. baicalensis* sponges and cell cultures of primmorphs (Chernogor et al., 2013). These unicellular eukaryotes produce a significant amount of carbohydrates, chlorophyll, fatty acids, and secondary metabolites (Latyshev et al., 1992; Bil et al., 1999). This Symbiosis provides mutual benefits of photosynthesis, as oxygen and nutrients pass from algae to the sponge and carbon dioxide and phosphate pass from sponge to algae (Wilkinson & Garrone, 1980; Pita et al., 2018).

We detected a mass death of green symbionts (*Chlorophyta*) in the diseased group and a shift in the microbial communities of sponges/primmorphs characterized by an increase in the abundance of phyla *Bacteroidetes* and *Proteobacteria*, *Flavobacteriaceae* and *Burkholderiaceae*. We observed an increase in the relative abundance of *Flavobacteriaceae* in the diseased sponges and especially in cultures of infected primmorphs. These bacteria are likely to be pathogens for healthy Baikal sponges. Members of the genus *Flavobacterium* include opportunistic pathogens common in aquatic systems (Chen et al., 2017; Kinnula, Mappes & Sundberg, 2017). Some species of *Flavobacterium* contain proteolytic and collagenolytic enzymes (Kharade & McBride, 2014; Nakayama et al., 2016). In addition, *Flavobacteriaceae* and *Cryomorphaceae* are associated with white band disease of corals (Gignoux-Wolfsohn & Vollmer, 2015; Certner & Vollmer, 2015). Species of the *Cytophaga*-*Flavobacterium* group cause diseases in marine ecosystems (Dobretsov et al., 2007; Romero et al., 2010; Certner & Vollmer, 2015).

Our results show an increase in the relative abundance of the families *Flavobacteriaceae* and *Burkholderiaceae* in the diseased sponges/primmorphs. The family *Burkholderiaceae* is characterized by the presence of ecologically extremely diverse organisms and contains environmental saprophytic organisms, phytopathogens, and opportunistic pathogens, including those common for freshwater ecosystems (Coenye, 2014).

Such stressful changes in the composition of sponge microbiomes are possibly related to environmental factors of Lake Baikal, such as temperature increase, changes in the warming tendencies of surface water layers, and an increase in vertical heat exchange (Smirnov et al., 2014; Troitskaya et al., 2015). Other include eutrophication in the coastal zone of the Lake Baikal (Timoshkin et al., 2016; Bondarenko et al., 2019).

A similar response to thermal stress in the bacterial biosphere and sponge-microbe associations occurs in marine environments (Simister et al., 2012a; Simister et al., 2012b; Erwin et al., 2012).

Imbalances in the microbial communities of sponges and model cell cultures of primmorphs might reflect several different opportunistic bacteria, including colonizing pathogens of diseased tissues (Price et al., 2017). We observed bio-cake layers containing different bacteria in model cultures of primmorphs infected by diseased sponges during the month of cultivation (Fig. 5D). These biofouling organisms can disrupt sponge functioning (Alexander et al., 2015). This phenomenon is similar to the jointly coordinated interactions of many bacteria species, which leads to the formation of layers of bio-cake on the artificial membranes (Waheed et al., 2017). A shift in the microbial composition from commensal bacterial symbionts to opportunistic species is likely to be a distinguishing feature of the Baikal sponge.

The primmorph system described in this work can be used to study the basic mechanisms of sponge disease development to expand our understanding of microbial relationships interactions with sponges endemic to Lake Baikal.

## Additional Information and Declarations

### Data Availability

The following information was supplied regarding data availability: Raw pyrosequencing data are available in NCBI: PRJNA480187.
